# Sets of RNA Repeated Tags and Hybridization-Sensitive Fluorescent Probes for Distinct Images of RNA in a Living Cell

**DOI:** 10.1371/journal.pone.0013003

**Published:** 2010-09-27

**Authors:** Takeshi Kubota, Shuji Ikeda, Hiroyuki Yanagisawa, Mizue Yuki, Akimitsu Okamoto

**Affiliations:** 1 RIKEN Advanced Science Institute, Saitama, Japan; 2 Precursory Research for Embryonic Science and Technology (PRESTO), Japan Science and Technology Agency, Saitama, Japan; University of Helsinki, Finland

## Abstract

**Background:**

Imaging the behavior of RNA in a living cell is a powerful means for understanding RNA functions and acquiring spatiotemporal information in a single cell. For more distinct RNA imaging in a living cell, a more effective chemical method to fluorescently label RNA is now required. In addition, development of the technology labeling with different colors for different RNA would make it easier to analyze plural RNA strands expressing in a cell.

**Methodology/Principal Findings:**

Tag technology for RNA imaging in a living cell has been developed based on the unique chemical functions of exciton-controlled hybridization-sensitive oligonucleotide (ECHO) probes. Repetitions of selected 18-nucleotide RNA tags were incorporated into the mRNA 3′-UTR. Pairs with complementary ECHO probes exhibited hybridization-sensitive fluorescence emission for the mRNA expressed in a living cell. The mRNA in a nucleus was detected clearly as fluorescent puncta, and the images of the expression of two mRNAs were obtained independently and simultaneously with two orthogonal tag–probe pairs.

**Conclusions/Significance:**

A compact and repeated label has been developed for RNA imaging in a living cell, based on the photochemistry of ECHO probes. The pairs of an 18-nt RNA tag and the complementary ECHO probes are highly thermostable, sequence-specifically emissive, and orthogonal to each other. The nucleotide length necessary for one tag sequence is much shorter compared with conventional tag technologies, resulting in easy preparation of the tag sequences with a larger number of repeats for more distinct RNA imaging.

## Introduction

Ribonucleic acid (RNA) is one of many essential biomolecules responsible for the expression, maintenance, and control of cell functions. The timing of mRNA expression is a significant factor in development or differentiation, as well as in the usual cellular cycle [1]–[3]. Imaging the behavior of RNA in a living cell is a powerful means for understanding RNA functions and acquiring spatiotemporal information in a single cell [4]–[6]. However, in contrast to the stable DNA double helix structure, RNA is highly diverse in terms of size, structure, mass, function, location, and expression timing, as well as in its sequences. The structural diversity of RNA especially plays an important role in characterizing the function of RNA, but it often makes fluorescence labeling of RNA difficult. An effective method to fluorescently label RNA regardless of the diversity of RNA is now required for spatiotemporal RNA imaging in a living cell. In addition, the labeling method with different colors for different RNA may make it easier simultaneously to analyze plural target RNA strands in a cell.

In this paper, we report sets of RNA tags and hybridization-sensitive fluorescent probes to obtain distinct images of RNA in a living cell. A compact and repeated label has been designed for sensitive detection of mRNA based on the chemistry of hybridization-sensitive fluorescence probes. The new tag technology enabled us to monitor clearly the target RNA expressing in a living cell.

## Materials and Methods

### Probe synthesis

The probes were synthesized through the phosphoramidite DNA synthesis according to the protocols reported previously [7]. The 2′-*O*-methyl ribonucleoside phosphoramidites were used except for the phosphoramidites of the doubly dye-labeled 2′-deoxyribonucleosides, D_514_ and D_640_. The product was purified by applying to a reverse-phase HPLC on a 5-ODS-H column (10 mm×150 mm, elution with a solvent mixture of 0.1 M triethylammonium acetate, pH = 7.0, linear gradient over 30 min from 5 to 30% acetonitrile at a flow rate of 3.0 mL/min), and then identified with MALDI–TOF mass spectrometry (here, the molecular weights of the two counter anions of dyes are not included in the value of M): ***anti*** **-gau-D_514_** CUUAUUCD_514_CAAUCCAAUC, calculated for C_239_H_303_N_65_O_128_P_17_S_2_ [M – H]^+^ 6725.1, found 6727.9; ***anti*** **-ggc-D_640_** CCUCD_640_CCUUGUUCCUGCC, calculated for C_240_H_307_N_59_O_133_P_17_S_2_ ([M – H]^+^) 6737.0, found 6739.9; ***anti*** **-aga-D_514_** CAAAUCCD_514_CUCAACCUCU, calculated for C_239_H_305_N_67_O_126_P_17_S_2_ ([M – H]^+^) 6723.0, found 6725.3.

### Photophysical properties

Absorption and fluorescence spectra of probes (0.5 µM) were measured with spectrophotometers UV2550 and RF-5300PC (Shimadzu), respectively, in a HEPES buffer (5 mM NaCl, 120 mM KCl, 25 mM HEPES, pH 7.2–KOH) using a quartz cuvette with a 1-cm path length. The fluorescence quantum yield of probes (0.1 µM) was measured using an absolute photoluminescence quantum yield measurement system, C9920-02 (Hamamatsu), equipped with an integrating sphere [8].

### Melting temperature

The melting temperatures (*T*_m_) of tag–probe duplexes (0.5 µM) were recorded in a HEPES buffer. The absorbance of the duplex samples at 260 nm was monitored from 10 to 90°C at a heating rate of 0.5°C/min. First derivatives were calculated from these profiles to determine the *T*_m_ values.

### Plasmid construction

A plasmid vector containing a fluorescent protein-coding region and a 64-time repeated tag sequence was synthesized as follows. As an example, an HcRed1-coding region was amplified from pHcRed1 (Clontech) by PCR using primers containing four-time repeated Tag(gau) sequences and *Xho*I, *Sal*I, and *Eco*RI restriction sites (forward, 5′-AAAAAGCAGGCTTCGAAGGAGATAGAACCATGGTGAGCGGCC-3′; reverse, 5′-AGAAAGCTGGGTGAATTCAGAGGTCGACCTTATTCTCAATCCAATCCTTATTCTCAATCCAATCCTTATTCTCAATCCAATCCTTATTCTCAATCCAATCCTCGAGTCAGTTGGCCTTCTCGGG-3′). The product was inserted into a pDONR221 vector by the BP reaction using the Gateway cloning technique (Invitrogen). The number of repeats was amplified by ligation and digestion with compatible enzymes. Then, the gene was transferred to an expression vector containing human cytomegalovirus (CMV) promoter (pT-REx DEST30, Invitrogen) by the LR reaction using the Gateway technique. The vector was purified with plasmid mini prep kit (Promega), dissolved in sterilized water, and stored at –30°C. The concentration was calculated from the absorbance at 260 nm (NanoDrop ND-1000, Thermo). Plasmids, pDsRed2-mito-Tag(ggc) ×64 and pmTFP1-mito-Tag(aga) ×64, were also constructed in the same way from pDsRed2-mito (Clontech) and pmTFP1-mitochondria (Allele Biotech) with each specific primer. The final construct has a tag region of 1264 nucleotides within 3′-UTR.

Human cDNAs of PSP1, SC35, and PML were purchased from Promega (Flexi ORF clone, Kazusa ORFenome Project [9]) as forms of Flexi vectors. A Fusion protein expression vector of the pmDsRed-PSP1 was constructed by inserting the PCR product of the PSP1 cDNA after the mDsRed coding region. A pSC35-DsRed2 was constructed by inserting the SC35 PCR product before the DsRed2 coding region. A pmDsRed-PML was constructed by using the In-Fusion PCR cloning technique (Clontech) from the PML PCR product and a linearized pmDsRed plasmid. The mDsRed and the DsRed2 vectors were prepared from pDsRed-monomer-actin and pDsRed2-mito (Clontech) by digestion, respectively.

### Cell culture

HeLa cells, gifted from Dr. Shinichi Nakagawa (RIKEN Advanced Science Institute), were grown in Dulbecco's modified Eagle's medium (DMEM) that was supplemented with serum (10% fetal bovine), penicillin (50 units/mL), and streptomycin (50 mg/mL). The cultures were incubated in a humidified atmosphere (5% CO_2_) at 37°C. For experimental use, cells (passage numbers 5–9) were cultured in glass-bottom dishes (Matsunami). Before microscope observation, the culture medium was washed and exchanged to an imaging medium (phenol red-free DMEM containing the serum and antibiotics). To make transcriptionally dysfunctioned cells, an RNA polymerase II inhibitor, α-amanitin (50 µg/mL), was added to the medium and incubated for 5 h before use.

### Live cell imaging

The cells were maintained at 37°C and 5% CO_2_ in an incubation system, INU (Tokai Hit), and monitored for several hours. Images were acquired with a motorized inverted microscope (Axio Observer Z1, Zeiss) equipped with a 63× objective (PlanApochromat NA 1.4, oil immersion) and an EM-CCD camera (evolve, Roper). The acquired images were analyzed and processed with operation software (AxioVision, Zeiss). Fluorescent probes were excited with a xenon arc lamp and the fluorescence was collected with an appropriate filter set (a yellow-green filter set, Ex 500/24–25, DM 520, Em 542/27–25; a red filter set, Ex 575–625, DM 645, Em 660–710; a cyan filter set, Ex 436/25, DM 455, Em 480/40; an orange filter set, Ex 545/25, DM 570, Em 605/70). Microinjection of probes and plasmid vectors was performed using a pneumatic injector (FemtoJet express, Eppendorf) with glass needles (FemtoTip, Eppendorf) and 3-D manipulators (Narishige).

Cells transfected with pmDsRed-PSP1, pSC35-DsRed2, or pmDsRed-PML were prepared using FuGene (Roche) according to the manufacturer's instructions. Nuclear localization images were acquired after 18–24 h from the transfection with a confocal unit (LSM 510, Zeiss). DsRed2, mDsRed, and D_514_ probes were excited with a He–Ne laser (543 nm) or an Ar laser (514 nm), and fluorescence images were taken through a 615 nm long-pass filter for DsRed2 and mDsRed, and a 520–550 nm band-pass filter for the D_514_ probe. Images were processed with the operation software and ImageJ software.

## Results

### Design of tag sequences and tag-binding probes

One of the effective labeling methods for biomolecules is fluorescence labeling to ‘tag’ sequences. A sequence independent of the original structure and function of the biomolecule's main part, the ‘tag’, is attached repeatedly at the end of the biomolecule we want to monitor. The larger the repetition number of the tag sequence, the stronger and clearer the fluorescence signal. Several labeling methods using tag sequences have been developed for the highly sensitive detection of biomolecules, such as conventional polyhistidine tags [10]–[13], tetracysteine tags in FlAsH technology [14]–[16], and tetraaspartate tags bound by multinuclear Zn(II) complexes [17]–[19] for protein labeling, and MS2 RNA [20]–[23], dye aptamer RNA [24]–[28], and RNA sequences in molecular beacon technology [29], [30] for nucleic acid labeling.

Hybridization-sensitive fluorescent DNA probes may serve as an effective and simple technology for tag labeling of nucleic acids, because they emit fluorescence only when they bind to the complementary sequences. A new type of hybridization-sensitive fluorescent probe, which has a doubly fluorescence-labeled nucleotide to achieve high fluorescence intensity for a hybrid with the target nucleic acid and effective quenching for a single-stranded state, has been reported [7], [31]. An excitonic interaction produced by the formation of an H-aggregate between dyes results in the suppression of fluorescence emission from the free probe. On the other hand, hybridization with the complementary strand shows a strong emission, because dissociation of dye aggregates by hybridization with the complementary nucleic acid results in the disruption of excitonic interaction. Such exciton-controlled hybridization-sensitive oligonucleotide (ECHO) probes are effective for live cell RNA imaging [32]–[34], and they can show many colors by varying the cyanine dye components [35].

Based on the knowledge of ECHO probes, we designed tag sequences for highly sensitive RNA imaging. The requirements for effective tag sequences are (i) sequence length appropriate for higher duplex stability but smaller tag size for synthesis of compact repeated tags; (ii) no formation of higher-ordered structures; (iii) no interference to the sequence, structure, and function of RNA main parts; and (iv) avoidance of self-dimerization of complementary ECHO probes. In addition, choosing several candidates for the tag sequences that are orthogonal to each other is desirable for multicolor tag labeling of plural target RNA strands.

**Figure 1:**
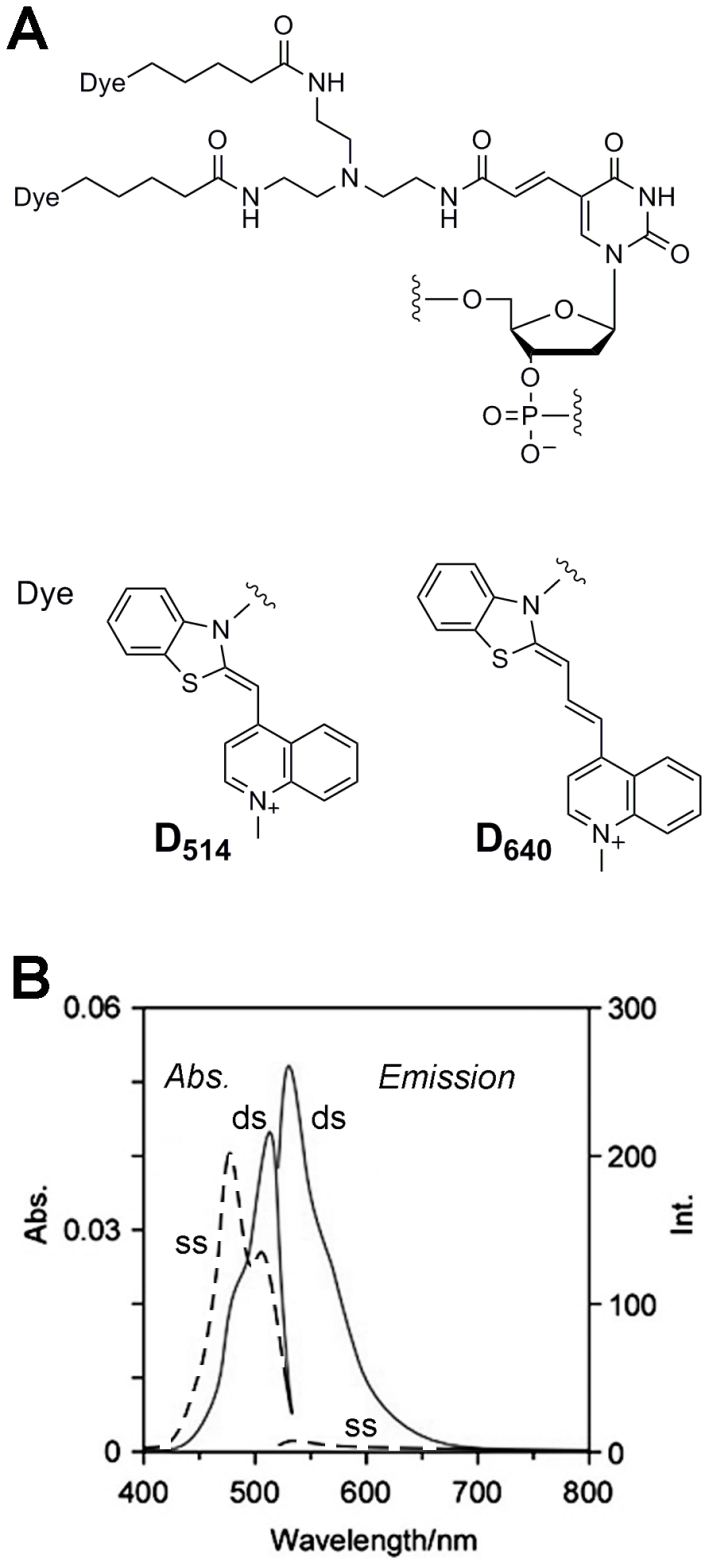
Structure and photophysical properties of ECHO probes. (A) Molecular structure and photophysical properties of D_514_ and D_640_ ECHO probes. (B) Typical absorption and fluorescence spectra of ECHO probes. The spectra of a single-stranded ***anti*** **-gau-D_514_** (ss) and the hybrid with the complementary RNA (ds) were measured in a HEPES buffer (120 mM KCl, 5 mM NaCl, 25 mM HEPES, pH = 7.2). Emission spectra were obtained at 513 nm.

We initially extracted 30,000 18-nucleotides (nt) sequences from the 200,000-nt sequences of naturally existing genome DNA strands based on the principal rule of avoiding self-dimerization of hybridization-sensitive probes, as mentioned in the reported papers [31]. After randomly extracting 300 18-nt sequences among them, we selected 20 18-nt sequences by considering whether the candidates include moderately mixed sequences, whether they still avoid the formation of any higher-ordered structures even if the sequence is repeated, and whether they are under orthogonal relationships for avoidance of interference between them. We next prepared these tag RNA sequences and the complementary ECHO probes (D_514_ or D_640_, Figure 1), and confirmed the sensitivity and orthogonality of the fluorescence emission. Finally, three 18-nt sequences that satisfied the condition described above for good hybridization-selective fluorescence emission were selected as tag RNA sequences (Table 1, Figure S1). The ECHO probes containing D_514_ showed very effective switching of fluorescence emission. Although the fluorescence switching of a D_640_ probe was less efficient as reported earlier (35), D_640_ was used as another fluorescence color because the excitation and emission wavelength of D_640_ do not overlap those of D_514_ probes and fluorescent proteins used in this study and the background fluorescence from the nonhybridized D_640_ probe is not so high. In this study, the 2′-*O*-methyl RNA backbone was adopted for the ECHO probes to gain nuclease resistance for long-term observation in cells, except for the 2′-deoxyribonucleotide of D_514_ and D_640_.

**Table 1:** Sequences and photophysical properties of tag RNA strands and ECHO probes. a

Sequences (5′→3′)	*T*_m_/°C b	λ_max_ (ε)/nm		λ_em_ (λ_ex_)/nm		Φ_f_ c	*I*_hybrid_/*I*_probe_ d
***anti*** **-gau-D_514_** CUUAUUC**D_514_**CAAUCCAAUC	–	478507	(86,600)(58,600)	535	(518)	0.04	–
+ Tag(gau) GAUUGGAUUGAGAAUAAG	69	513	(90,800)	532	(518)	0.52	36
***anti*** **-ggc-D_640_** CCUC**D_640_** CCUUGUUCCUGCC	–	581	(170,000)	656	(640)	0.04	–
+ Tag(ggc) GGCAGGAACAAGGAGAGG	79	581	(134,400)	660	(650)	0.06	4
***anti*** **-aga-D_514_** CAAAUCC**D_514_**CUCAACCUCU	–	477505	(106,600)(58,200)	538	(518)	0.02	–
+ Tag(aga) AGAGGUUGAGAGGAUUUG	73	512	(103,200)	534	(517)	0.34	80

*^a^*ECHO probes consisted of 2′-*O*-methyl ribonucleotides except for D_514_ and D_640_ 2′-deoxyribonucleotide.

*^b^*All *T*_m_ values of the tag–probe hybrids (500 nM) were obtained in a HEPES buffer (120 mM KCl, 5 mM NaCl, 25 mM HEPES, pH = 7.2).

*^c^*Fluorescence quantum yield measured using 100 nM probe in the HEPES buffer on excitation at 480 nm for ***anti*** **-gau-D_514_** and ***anti*** **-aga-D_514_** and at 600 nm for ***anti*** **-ggc-D_640_**.

*^d^*Ratio of the fluorescence intensities of the tag–probe hybrids and probe alone at the λ_em_ of hybrids.

### Construction of plasmids

The advantage of tag technology is that our designed tag sequence can be incorporated repeatedly into the mRNA end to obtain strong fluorescence intensity and clear fluorescence images. In this study, we prepared plasmid vectors containing a 64-time tag-repeated sequence. The repeated tag sequence is attached to a sequence end part, e.g., 3′-untranslated region (UTR) of the target RNA, which has a minor influence on the RNA structures and functions. The tag-attached mRNA sequences of fluorescent proteins HcRed1 (λ_max_ = 618 nm), DsRed2-mito (λ_max_ = 583 nm), and mTFP1-mito (λ_max_ = 492 nm) were prepared using the expression plasmid vectors containing a CMV promoter region. We synthesized in advance a vector containing a four-time tag-repeated sequence that lies between *Xho*I (C∧TCGAG) and *Sal*I (G∧TCGAC) enzymatic cleavage sites (Figure S2). An eight-time tag-repeated sequence was next obtained by insertion of the *Xho*I/*Eco*RI small fragment into the *Sal*I/*Eco*RI large fragment. Three more cycles of cleavage and ligation provided a plasmid containing a 64-time tag-repeated sequence (total 1264 nucleotide length including digestion sequences) at the 3′-UTR of mRNA.

### Fluorescence imaging

**Figure 2:**
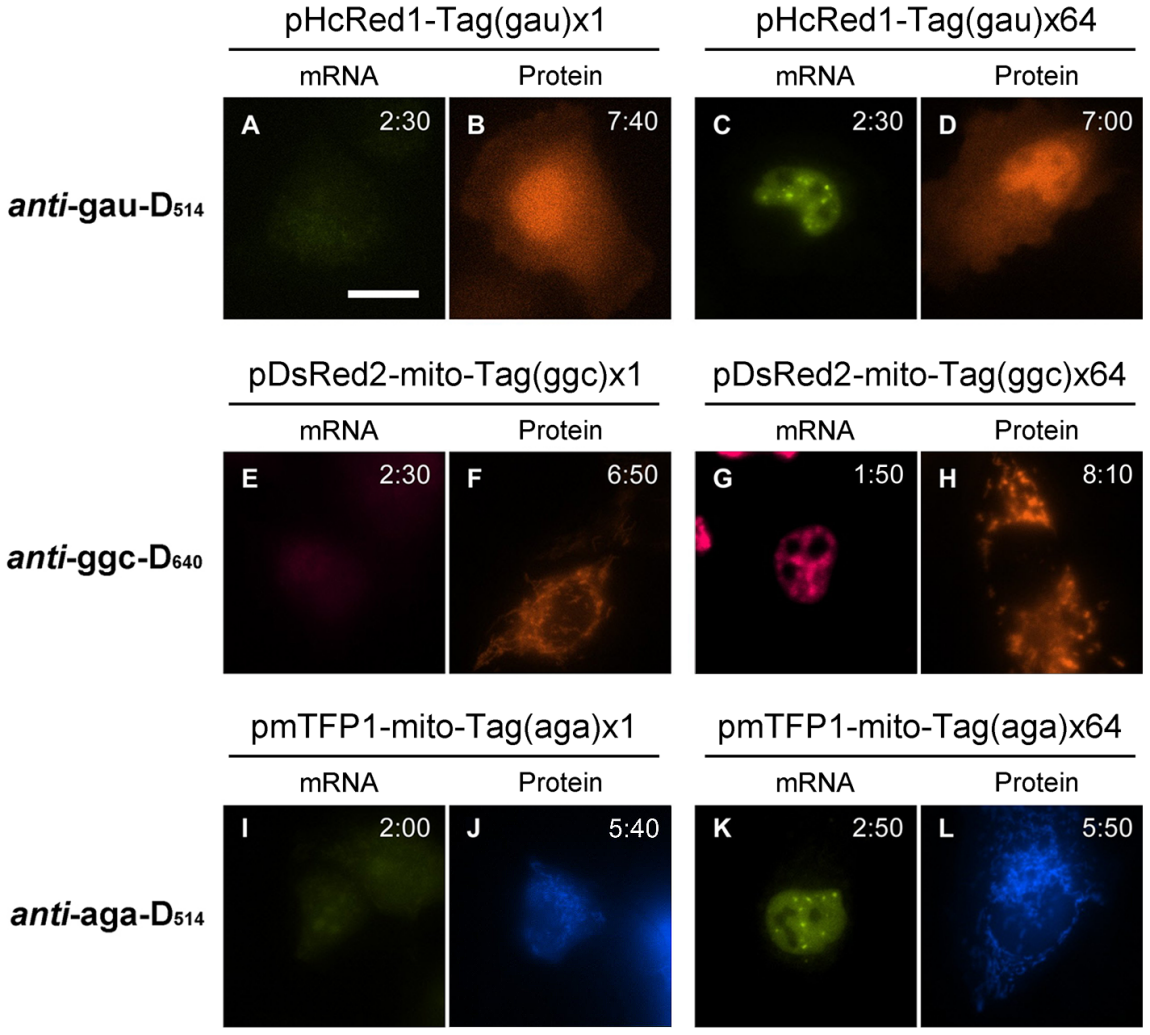
Imaging of expressed mRNA in living HeLa cells. Mixtures of ECHO probe (10 µM) and fluorescent protein-encoding plasmid (50 ng/µL) in sterilized water were microinjected into a living HeLa cell. Images were acquired every 10 min for 9 h after microinjection, the acquisition times from the onset being displayed in each image (hh:mm). The probe fluorescence was collected with a yellow-green filter set (Ex 500/24–25, DM 520, Em 542/27–25) for ***anti*** **-gau-D_514_** and ***anti*** **-aga-D_514_** (A, C, I, and K) and a red filter set (Ex 575–625, DM 645, Em 660–710) for ***anti*** **-ggc-D_640_** (E and G). Fluorescence from fluorescent proteins was collected with an orange filter set (Ex 545/25, DM 570, Em 605/70) for HcRed1 and DsRed2-mito (B, D, F, and H) and a cyan filter set (Ex 436/25, DM 455, Em 480/40) for mTFP1-mito (J and L). Scale bar, 20 µm.

The mixture of a tag-attached plasmid and a corresponding ECHO probe was microinjected into a HeLa cell nucleus and cell images acquired over 8 h (Figure 2). In the case that a plasmid pHcRed1-Tag(gau) ×64 (50 ng/µL) and the corresponding D_514_ ECHO probe ***anti*** **-gau-D_514_** (10 µM) were microinjected, fluorescence emission in the nucleus was observed in 2 h after microinjection (C, *n* = 13). After a further 5 h, fluorescence with a longer wavelength originating from HcRed1 fluorescent protein appeared (D). The first fluorescence emission originated from the in-cell hybridization of the probe ***anti*** **-gau-D_514_** with the tag sequence of the expressed mRNA. The existence of plasmid DNA showed no influence on the fluorescence intensity of the probe, and only the expressed RNA can make the complementary fluorescence probe shine (Figure S3). Another plasmid–probe pair with different colors of expressed products, pDsRed2-mito-Tag(ggc) ×64 (50 ng/µL) and ***anti*** **-ggc-D_640_** (10 µM), also showed fluorescence emission in a nucleus originating from DsRed2 mRNA expression (G, *n* = 7) and then the fluorescence of DsRed2-mito fluorescent protein after 8 h (H). The microinjection of pmTFP1-mito-Tag(aga) ×64 (50 ng/µL) and ***anti*** **-aga-D_514_** (10 µM) also resulted in the appearance of fluorescence, including several fluorescent puncta in a cell nucleus (K, *n* = 6) and then fluorescence in the cytoplasm (L).

Repeated tag sequences produce cell images with sharper fluorescence. When we used a plasmid that includes only one tag, e.g., pHcRed1-Tag(gau) ×1, the fluorescence of ***anti*** **-gau-D_514_** in the cell was less sharp (A, *n* = 9). The fluorescence intensity of expressed HcRed1 fluorescent protein (B) was at almost the same level as that from pHcRed1-Tag(gau) ×64 (D). The repetition number of the tag attached to 3′-UTR did not influence the efficiency of protein expression but did affect the sharpness of the fluorescence images of the expressed mRNA. An indistinct fluorescence image in mRNA imaging was also observed when other plasmids including a single tag sequence were used, pDsRed2-mito-Tag(ggc) ×1 (E, *n* = 8) and pmTFP1-mito-Tag(aga) ×1 (I, *n* = 10).

Fluorescence emission from ECHO probes reflects the expression of the target mRNA (Figure S4). When the mixture of pHcRed1-Tag(gau) ×64 and ***anti*** **-gau-D_514_** was injected into the HeLa cells that had been incubated with an RNA polymerase II inhibitor α-amanitin [36], [37] (50 µg/mL) for 5 h, we observed almost no fluorescence emission from mRNA or protein, and the images were quite different from those of the cells without treatment by α-amanitin. The fluorescence intensity of α-amanitin-treated cells was similar to that in the cell to which the probe was added but the plasmid was not added. The expression-sensitive fluorescence emission suggests that the Tag(gau) sequence of expressed mRNA was labeled by binding of the fluorescent probe ***anti*** **-gau-D_514_**.

**Figure 3:**
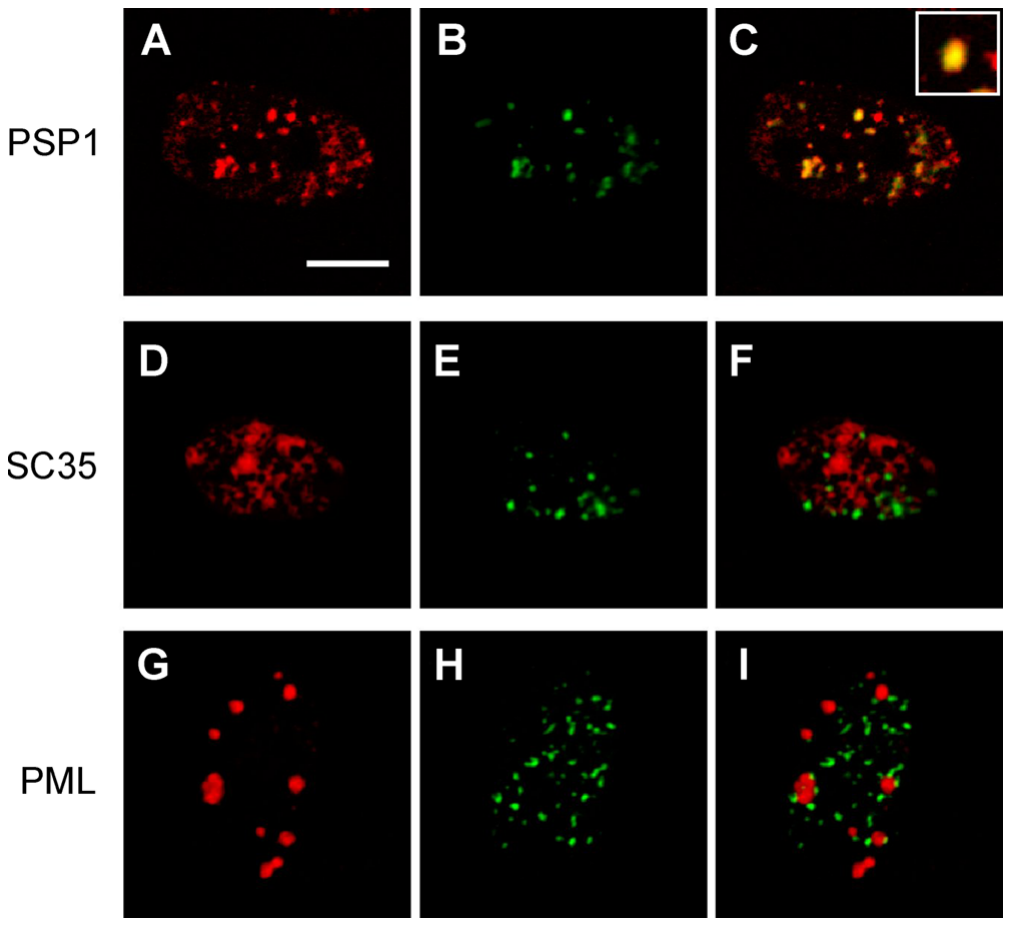
Fluorescent puncta in the nucleus of a HeLa cell. Images were acquired at 1 h after microinjection of ***anti*** **-aga-D_514_** (10 µM) and pmTFP1-mito-Tag(aga) ×64 (50 ng/µL). Scale bar, 10 µm. (A–C) The cell nucleus including fluorescence-labeled PSP1. (A) Fluorescence from expressed mDsRed-PSP1, (B) fluorescence from the hybrid of ***anti*** **-aga-D_514_** and expressed mRNA, and (C) the merged image (inset, a magnified figure of one of the overlapping fluorescent puncta). (D–F) The cell nucleus including fluorescence-labeled SC35. (D) Fluorescence from expressed SC35-DsRed2, (E) fluorescence from the hybrid of ***anti*** **-aga-D_514_** and expressed mRNA, and (F) the merged image. (G–I) The cell nucleus including fluorescence-labeled PML. (G) Fluorescence from expressed mDsRed–PML, (H) fluorescence from the hybrid of ***anti*** **-aga-D_514_** and expressed mRNA, and (I) the merged image.

Several fluorescent puncta were observed in a cell nucleus by addition of ECHO probes and expression of 64-time tag-repeated mRNA into a living HeLa cell. They varied in number, size, shape, and fluorescence intensity in each cell and at each time point, and distributed over other than the nucleolus area that was clearly visualized with differential interference contrast observation (Figure S5). Their punctate nuclear localization was derived from emission of ECHO-labeled mRNA binding with PSP1, one of the paraspeckle proteins in nuclei, which have been reported to be irregularly shaped subcellular compartments with diameters of 0.2–1.0 µm [38]–[40]. Microinjection of pmTFP1-mito-Tag(aga) ×64 and ***anti*** **-aga-D_514_** into the nuclei of the mDsRed-PSP1-expressed HeLa cells exhibited clear fluorescent puncta from ***anti*** **-aga-D_514_** within 1 h, and the punctate pattern overlapped the fluorescent puncta from mDsRed-PSP1 (Figure 3). Because fluorescence from ***anti*** **-aga-D_514_** was not observed in the absence of pmTFP1-mito-Tag(aga) ×64, the fluorescent puncta from ***anti*** **-aga-D_514_** overlapping the fluorescence from PSP1 in HeLa cell nuclei suggests that probe-binding mTFP1-mito mRNA localizes at PSP1. The punctate pattern of ***anti*** **-aga-D_514_** in the presence of pmTFP1-mito-Tag(aga) ×64 did not overlap the fluorescence from other nuclear speckles, SC35, or PML fused with a fluorescent protein.

### Multicolor imaging using orthogonal tags

**Figure 4:**
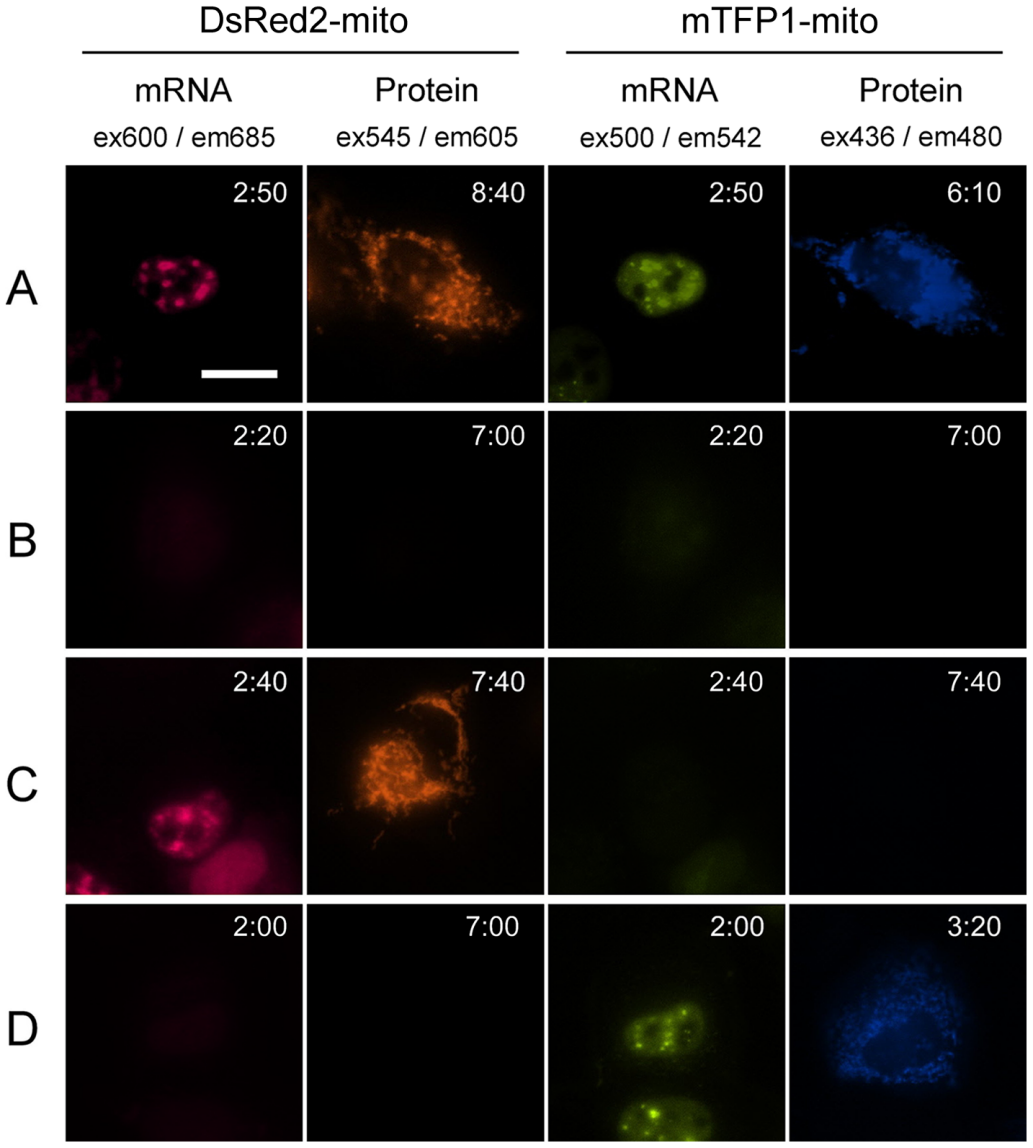
Simultaneous dual RNA imaging in a single HeLa cell. (A) Microinjection of a mixture of ***anti*** **-ggc-D_640_**, ***anti*** **-aga-D_514_**, pDsRed2-mito-Tag(ggc) ×64, and pmTFP1-mito-Tag(aga) ×64. Filter sets were used as described in Figure 2. (B) Microinjection of a mixture of ***anti*** **-ggc-D_640_** and ***anti*** **-aga-D_514_**. (C) Microinjection of a mixture of ***anti*** **-ggc-D_640_**, ***anti*** **-aga-D_514_**, and pDsRed2-mito-Tag(ggc) ×64. (D) Microinjection of a mixture of ***anti*** **-ggc-D_640_**, ***anti*** **-aga-D_514_**, and pmTFP1-mito-Tag(aga) ×64. Images were acquired every 10 min, the acquisition times being displayed in each image (hh:mm). Scale bar, 20 µm.

Establishment of a multicolor RNA imaging method makes it possible to simultaneously monitor the behaviors of different RNA sequences in a single cell. The orthogonal pairs of two different tag sequences and two differently colored ECHO probes, i.e., two tag–probe pairs that do not interfere with each other, would be useful for simultaneous multicolor RNA monitoring. For example, ECHO probes ***anti*** **-ggc-D_640_** and ***anti*** **-aga-D_514_** are available for simultaneous imaging because they have different fluorescence emission wavelengths (λ_em_, approximately 685 nm and 542 nm, respectively) and the fluorescent proteins mTFP1 and DsRed2 also have different emission wavelengths (λ_em_, approximately 605 nm and 480 nm, respectively). Simultaneous imaging of expression from two RNA molecules and two proteins becomes possible using four colors, which are separable with four optical filter sets (Figure. 4). Microinjection of the mixture of two probes, ***anti*** **-ggc-D_640_** and ***anti*** **-aga-D_514_**, and two plasmids, pDsRed2-mito-Tag(ggc) ×64 and pmTFP1-mito-Tag(aga) ×64, induced fluorescence emission in a nucleus at both emission wavelengths of the probes. Fluorescence from the expressed proteins was observed in the same cell after a few hours (A, *n*  = 7). Microinjection of two probes without any plasmids showed no significant fluorescence emission, except for very weak background fluorescence from the ECHO probes (B, *n* = 12). When two probes and one plasmid pDsRed2-mito-Tag(ggc) ×64 were microinjected, we observed fluorescence in a nucleus derived from ***anti*** **-ggc-D_640_** and orange fluorescence of mitochondria derived from DsRed2-mito, whereas there was little fluorescence from ***anti*** **-aga-D_514_**, suggesting that tag misrecognition is completely avoided and the orthogonality of the tag–probe pairs is maintained in the cell (C, *n* = 3). Similarly, microinjection of two probes and one plasmid pmTFP1-mito-Tag(aga) ×64 resulted in fluorescence emission in a nucleus derived from ***anti*** **-aga-D_514_** and blue fluorescence emission of mitochondria derived from mTFP1-mito, whereas there was little fluorescence from ***anti*** **-ggc-D_640_** (D, *n* = 4). The ECHO probes designed for the tag-labeling system recognized only their complementary tags attached to mRNA in living cells without disturbance of the other RNA tag. This RNA-labeling technology with orthogonal tag–probe pairs is available for simultaneous multicolor RNA detection.

## Discussion

ECHO probes are sequence-specific, hybridization-sensitive, quencher-free fluorescent probes for RNA detection, which have been designed using the concept of fluorescence quenching caused by the intramolecular excitonic interaction of fluorescent dyes [7], [31]–[35]. An excitonic interaction is produced by the formation of an H-aggregate of fluorescent dyes, which is observed as a blue shift of the absorption band of the dye in the absorption spectra (Figure 1B), and, as a result, emission from the doubly thiazole orange-labeled nucleoside incorporated into ECHO probes is suppressed before hybridization. Dissociation of dye aggregates and subsequent intercalation into the duplex structure caused by hybridization with the complementary RNA results in disruption of the excitonic interaction and strong emission from the hybrid. This probe achieved a large, rapid, reversible change in fluorescence intensity in sensitive response to the amount of target RNA, and facilitated spatiotemporal monitoring of the behavior of intracellular RNA.

The hybridization-sensitive fluorescence emission of ECHO probes is suitable for RNA imaging by means of tag technology. Several tag technologies have been developed for fluorescence imaging of intracellular RNA (Table 2). One of the well-known tag technologies for RNA detection is the MS2 system [20]–[23]. The RNA of interest is modified with multiple copies of the 19-nt MS2 stem–loop motifs in tandem and is detected by expressing MS2-coat protein fused to a fluorescent protein in the same cell. The MS2 system is suitable for one color label but unsuitable for simultaneous multicolor imaging of plural RNA sequences because of the lack of variety of tag sequences. In addition, the fused protein emits fluorescence that is independent of binding to the target RNA, thus the excess protein has to be sequestered in the nucleus owing to a nuclear localization signal. One of the well-known classes of fluorescent oligonucleotides for RNA detection is the molecular beacon [29], [30]. The fluorescence from the probe is usually controlled by Förster resonance energy transfer between dyes attached to the strand ends in the stem–loop DNA structure, and the probe emits fluorescence through the linearization of the probe structure by binding to the target RNA. In this technology, longer spacers are necessary between repeated tag sequences to avoid fluorescence quenching by the energy transfer between tag-binding probes. Another approach for live cell detection with a fluorescent dye-binding RNA aptamer has also been reported. Malachite green emits fluorescence when it binds to its aptamer RNA [24], [41]. An array of malachite green aptamers, which is attached to the end of the RNA of interest, acts as the tag for fluorescence labeling of RNA. Relatively longer RNA sequences are required for one tag, thus synthesis of a large number of tag repeats is difficult.

**Table 2:** Tag properties of RNA tag-labeling technologies.

Probes	Tag structure	Reversibility a	*n* b	Plasmid design c (Probe-binding length) d	*N* e	Ref. No.
ECHO	Linear	Yes	2	84 nt for 4 tags (18 nt)	128	–
MS2	Stem–loop	No	2	114 nt for 3 tags (19 nt)	96	23
Molecular beacon f	Linear	Yes	1	50 nt for 1 tag (24 nt)	96	30
Malachite green	(Aptamer)^g^	Yes	1	38–54 nt for 1 tag (38–54 nt)	5	24

*^a^*Switching of fluorescence emission depending on the existence of tag RNA.

*^b^*Number of dyes per one tag unit. Two dyes are attached to a nucleotide in an ECHO probe and two MS2-coat proteins bind to a tag RNA.

*^c^*Nucleotide length of a synthetic DNA unit containing one or more tag sequences for plasmid synthesis and the number of tag sequences therein.

*^d^*Nucleotide length necessary for probe binding in a tag sequence.

*^e^*Maximum repetition number of the tag attached to an RNA sequence reported in the literature.

*^f^*Molecular beacons form a linear structure at the emissive RNA-binding state and a stem–loop structure in the nonemissive free state.

*^g^*The structure of the malachite green aptamer includes two Watson-Crick helices flanking an asymmetric loop. See ref. [41].

Three pairs of an 18-nt RNA tag and the complementary ECHO probes in this study are highly thermostable (*T*_m_ approximately 70°C), sequence-specifically emissive, and orthogonal to each other. In addition, because a fluorescent nucleotide is usually incorporated into the center part of the probe, interference between the fluorescent dyes of probes is avoided even if the probes are arranged tandemly onto the repeated tag sequences. Therefore, one of the significant advantages of the ECHO probe in tag technology is that the probe does not require either a long tag sequence for fluorescent probe binding or a long spacer sequence to avoid interference between tag-binding probes. The nucleotide length necessary for one tag sequence is shorter, resulting in easy preparation of the tag sequences with a larger number of repeats. For example, the preparation of a 128-time tag-repeated plasmid, pmTFP1-mito-Tag(aga) ×128, is also possible. After microinjection of the plasmid and ***anti*** **-aga-D_514_**, a clear fluorescence image showing expression of the RNA in a HeLa cell was obtained (Figure S6). Although the lowest amount of mRNA, which can be detected by fluorescent probes in living cells, may depend on the performance of the microscopes and cameras used in the observation, the higher number of tag repetition will facilitate to obtain the sharper fluorescence image of a small amount of the expressed mRNA.

In conclusion, a tag technology for RNA imaging in a living cell has been developed based on the unique chemistry of ECHO probes. This is a new method for spatiotemporal monitoring of RNAs in cells by using pairs of the repeated 18-nt tags incorporated into the mRNA 3′-UTR and the complementary ECHO probes. The mRNA in a nucleus was detected clearly as fluorescent puncta, and the images of the expression of two mRNAs were obtained independently and simultaneously with two orthogonal tag–probe pairs. For ECHO probes, there may still remain further aspects to be examined toward an intracellular RNA analysis, such as detection of a very small amount of original endogenous RNA. However, the new tag technology would be a powerful tool for monitoring the behavior of the artificially expressed RNA in cells. We anticipate that highly sensitive imaging technology, supported by the new concept of RNA tags and photochemical probes, will be the starting point for the development of a practical assay for the detection of RNAs in living cells and monitoring of their spatiotemporal characteristics and interaction.

## Supporting Information

Figure S1 Absorption and fluorescence spectra of three ECHO probes. The spectra were measured in a HEPES buffer (120 mM KCl, 5 mM NaCl, 25 mM HEPES, pH = 7.2). Blue, single-stranded ECHO probes (500 nM); Red, ECHO probes hybridized with the complementary RNA. Emission spectra were obtained at each maximum excitation wavelength. (A) *anti*-gau-D_514_, (B) *anti*-ggc-D_640_, and (C) *anti*-aga-D_514_.
(2.05 MB TIF)

Figure S2 Schematic illustrations of a plasmid vector containing a repeated tag sequence. (A) An overview of the plasmid constitution. The plasmid contains a CMV promoter, a fluorescent protein-coding region, and a 3′-UTR with a 64-time tag-repeated sequence and an SV40 polyadenylation region. (B) Tag amplification processes. After preparing a plasmid containing a four-time tag-repeated sequence, the plasmid was digested with restriction enzymes ((a) a set of *Sal*I (G∧TCGAC) and *Eco*RI (G∧AATTC) or (b) a set of *Xho*I (C∧TCGAG) and *Eco*RI). Ligation at the two compatible ends and *Eco*RI site provided a plasmid containing an eight-time tag-repeated sequence. The reaction cycles were repeated further three times to gain a plasmid containing a 64-time tag-repeated sequence.
(2.05 MB TIF)

Figure S3 Tag-specific fluorescence of *anti*-gau-D_514_. (1) *anti*-gau-D_514_ (125 nM) in HEPES buffer on excitation at 514 nm (a blue line); (2) the mixture of *anti*-gau-D_514_ and the plasmid pHcRed1-Tag(gau) ×64 (1.25 ng/µL) (a green line overlapped by a blue line); (3) the mixture of *anti*-gau-D_514_, the plasmid and the complementary RNA (125 nM) (a red line). (Inset) Relative fluorescence intensities of the mixtures. The values were calculated from the intensities at 532 nm.
(2.76 MB TIF)

Figure S4 Fluorescence emission dependent on expression of mRNA in the nuclei of living HeLa cells. (A and B) Microinjection of a mixture of *anti*-gau-D_514_ (10 µM) and pHcRed1-Tag(gau) ×64 (50 ng/µL). (C and D) Microinjection of *anti*-gau-D_514_ (10 µM). (E and F) Microinjection of a mixture of *anti*-gau-D_514_ (10 µM) and pHcRed1-Tag(gau) ×64 (50 ng/µL) into α-amanitin-treated cells (50 µg/mL, 5 h). Images were collected with a yellow-green filter set (A, C, and E) for the fluorescence from *anti*-gau-D_514_ and with an orange filter set (B, D, and F) for the fluorescence from an expressed protein HcRed1. Images were acquired every 10 min, the acquisition times being displayed in each image (hh:mm). Scale bar, 20 µm.
(2.04 MB TIF)

Figure S5 Fluorescent puncta in the nucleus of a HeLa cell. Images were acquired at 2.5 h after microinjection of *anti*-gau-D_514_ (10 µM) and pHcRed1-Tag(gau) ×64 (50 ng/µL). (A) Differential interference contrast observation showing elipsoidal nucleus with three nucleolus. (B) Several fluorescent puncta showing probe-bound RNA. (C) A merged image. Scale bar, 10 µm.
(2.89 MB TIF)

Figure S6 Expression of the mRNA containing a 128-time tag-repeated sequence in living HeLa cells. (A) Fluorescence from *anti*-aga-D_514_ showing the expression of tag-attached mRNA from pmTFP1-mito-Tag(aga) ×128. (B) Fluorescence from mTFP1-mito. Scale bar, 20 µm.
(1.51 MB TIF)
