# Supplementary figures and images for: Sets of RNA Repeated Tags and Hybridization-Sensitive Fluorescent Probes for Distinct Images of RNA in a Living Cell
Source: PLoS One. 2010 Sep 27;5(9):e13003. doi: 10.1371/journal.pone.0013003 (PMC2946342; doi:10.1371/journal.pone.0013003)

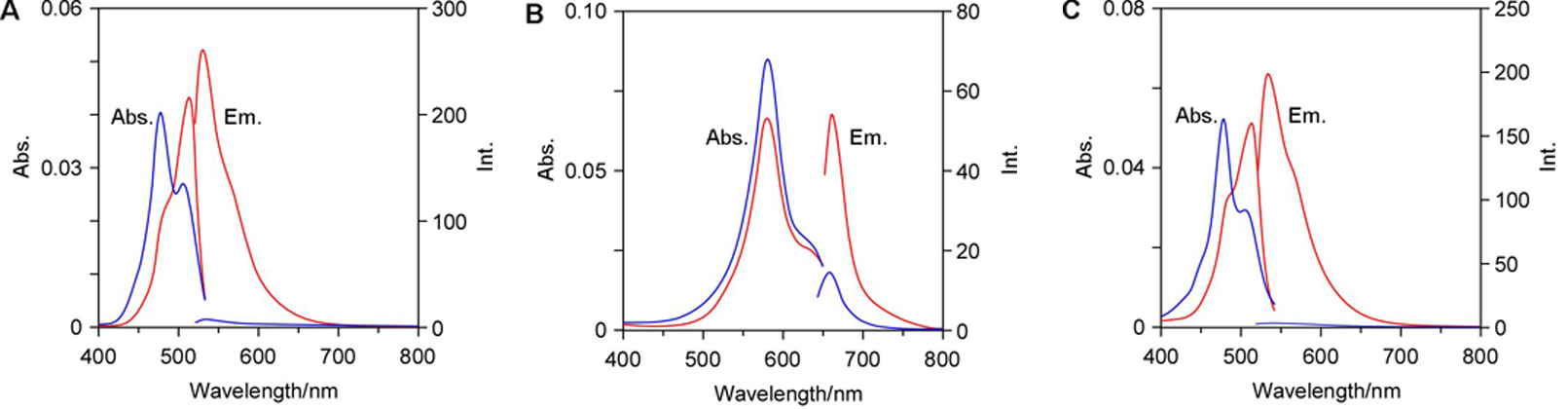

Supplement: Figure S1 — Absorption and fluorescence spectra of three ECHO probes. The spectra were measured in a HEPES buffer (120 mM KCl, 5 mM NaCl, 25 mM HEPES, pH = 7.2). Blue, single-stranded ECHO probes (500 nM); Red, ECHO probes hybridized with the complementary RNA. Emission spectra were obtained at each maximum excitation wavelength. (A) anti-gau-D514, (B) anti-ggc-D640, and (C) anti-aga-D514. (2.05 MB TIF) [file pone.0013003.s001.tif]

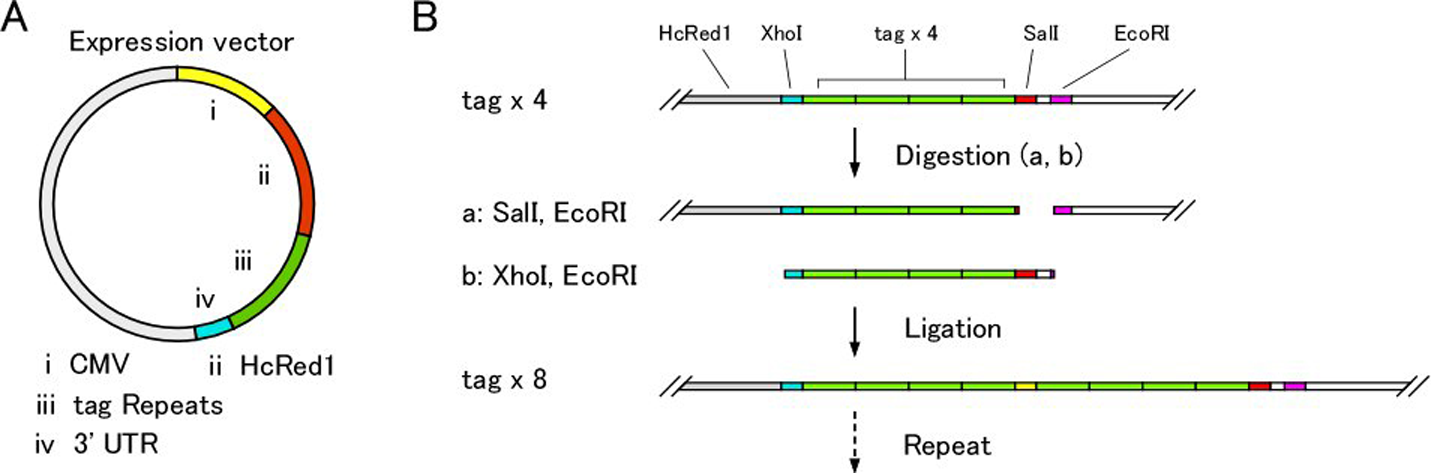

Supplement: Figure S2 — Schematic illustrations of a plasmid vector containing a repeated tag sequence. (A) An overview of the plasmid constitution. The plasmid contains a CMV promoter, a fluorescent protein-coding region, and a 3′-UTR with a 64-time tag-repeated sequence and an SV40 polyadenylation region. (B) Tag amplification processes. After preparing a plasmid containing a four-time tag-repeated sequence, the plasmid was digested with restriction enzymes ((a) a set of SalI (G∧TCGAC) and EcoRI (G∧AATTC) or (b) a set of XhoI (C∧TCGAG) and EcoRI). Ligation at the two compatible ends and EcoRI site provided a plasmid containing an eight-time tag-repeated sequence. The reaction cycles were repeated further three times to gain a plasmid containing a 64-time tag-repeated sequence. (2.05 MB TIF) [file pone.0013003.s002.tif]

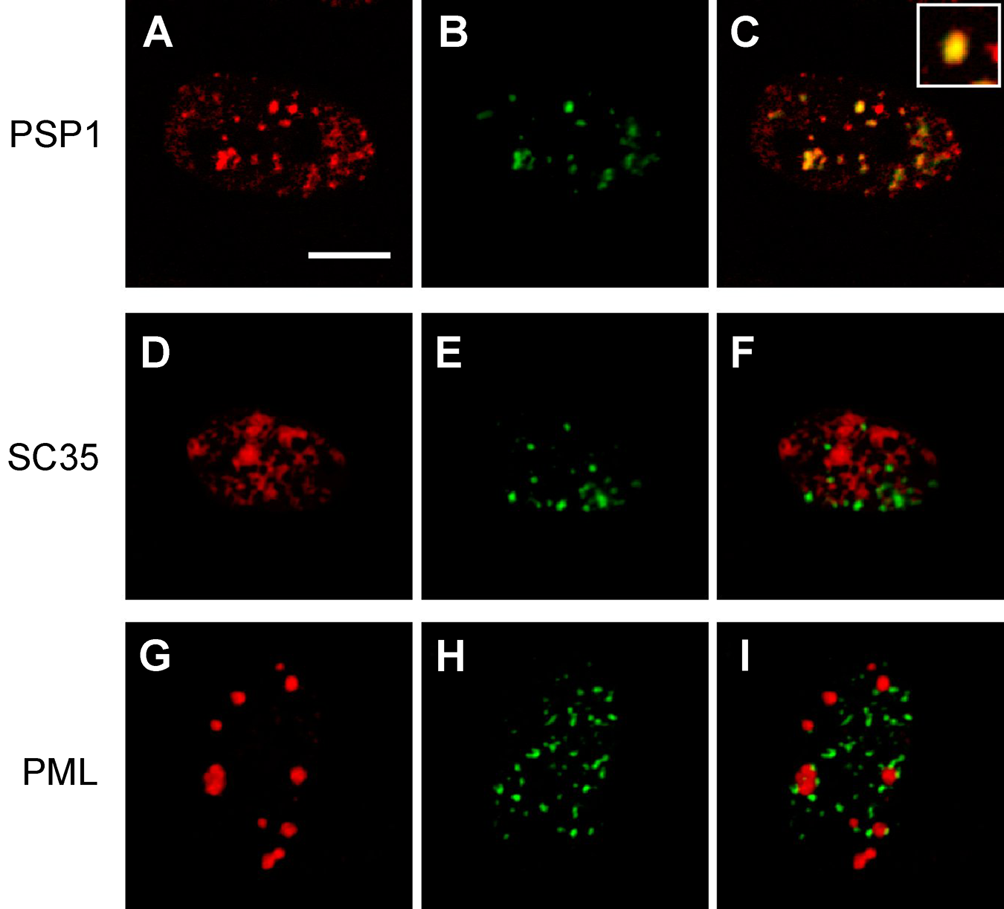

Supplement: Figure S3 — Tag-specific fluorescence of anti-gau-D514. (1) anti-gau-D514 (125 nM) in HEPES buffer on excitation at 514 nm (a blue line); (2) the mixture of anti-gau-D514 and the plasmid pHcRed1-Tag(gau) ×64 (1.25 ng/µL) (a green line overlapped by a blue line); (3) the mixture of anti-gau-D514, the plasmid and the complementary RNA (125 nM) (a red line). (Inset) Relative fluorescence intensities of the mixtures. The values were calculated from the intensities at 532 nm. (2.76 MB TIF) [file pone.0013003.s003.tif]

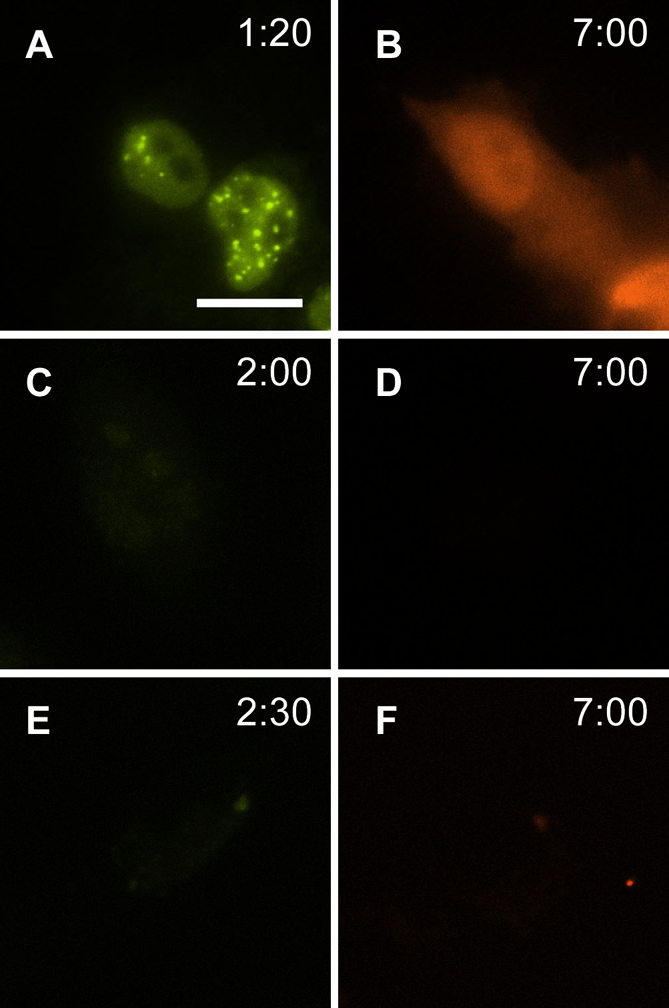

Supplement: Figure S4 — Fluorescence emission dependent on expression of mRNA in the nuclei of living HeLa cells. (A and B) Microinjection of a mixture of anti-gau-D514 (10 µM) and pHcRed1-Tag(gau) ×64 (50 ng/µL). (C and D) Microinjection of anti-gau-D514 (10 µM). (E and F) Microinjection of a mixture of anti-gau-D514 (10 µM) and pHcRed1-Tag(gau) ×64 (50 ng/µL) into α-amanitin-treated cells (50 µg/mL, 5 h). Images were collected with a yellow-green filter set (A, C, and E) for the fluorescence from anti-gau-D514 and with an orange filter set (B, D, and F) for the fluorescence from an expressed protein HcRed1. Images were acquired every 10 min, the acquisition times being displayed in each image (hh:mm). Scale bar, 20 µm. (2.04 MB TIF) [file pone.0013003.s004.tif]

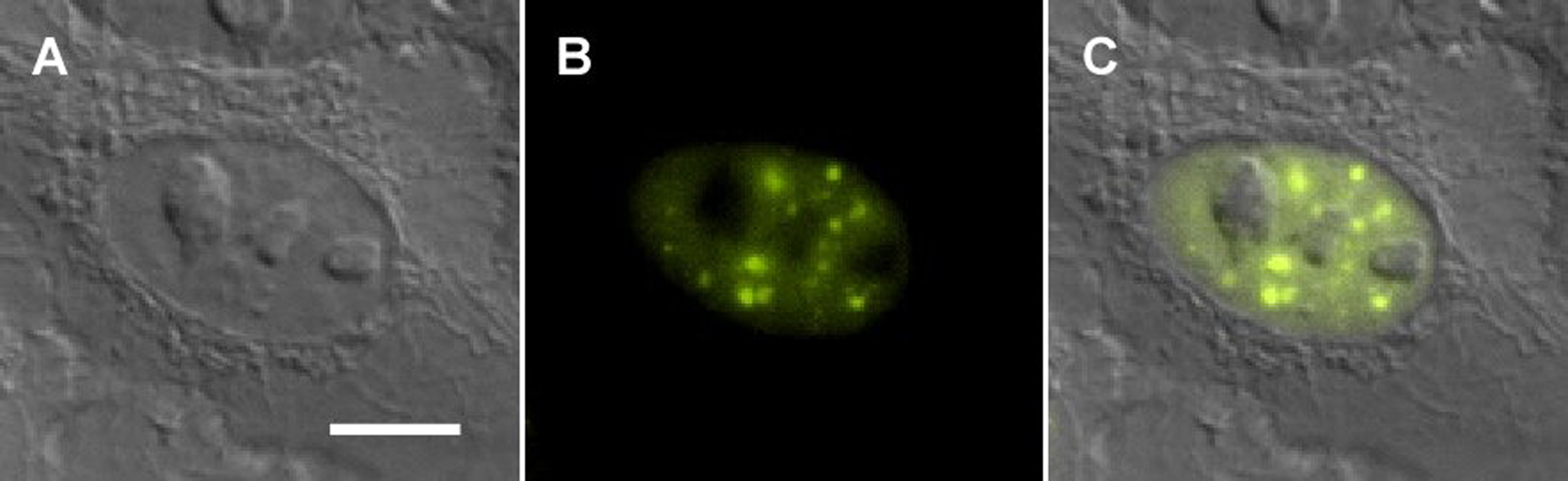

Supplement: Figure S5 — Fluorescent puncta in the nucleus of a HeLa cell. Images were acquired at 2.5 h after microinjection of anti-gau-D514 (10 µM) and pHcRed1-Tag(gau) ×64 (50 ng/µL). (A) Differential interference contrast observation showing elipsoidal nucleus with three nucleolus. (B) Several fluorescent puncta showing probe-bound RNA. (C) A merged image. Scale bar, 10 µm. (2.89 MB TIF) [file pone.0013003.s005.tif]

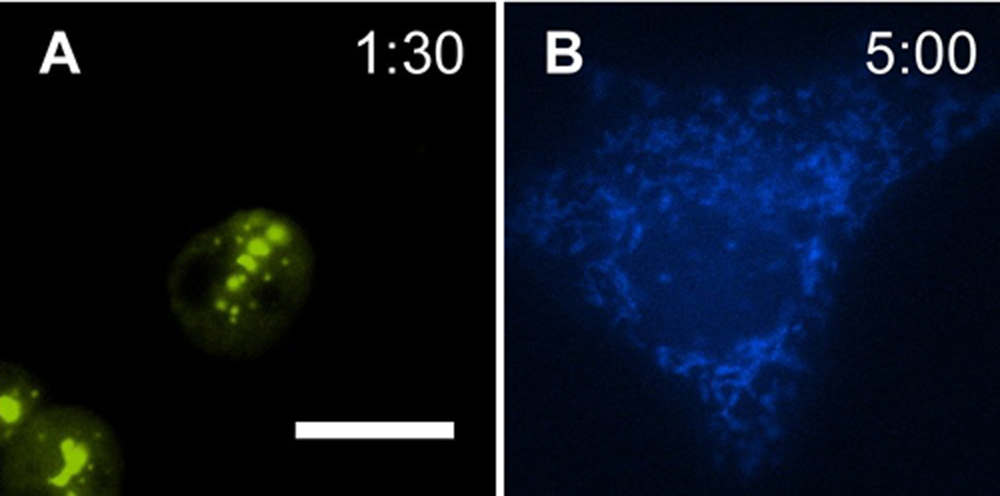

Supplement: Figure S6 — Expression of the mRNA containing a 128-time tag-repeated sequence in living HeLa cells. (A) Fluorescence from anti-aga-D514 showing the expression of tag-attached mRNA from pmTFP1-mito-Tag(aga) ×128. (B) Fluorescence from mTFP1-mito. Scale bar, 20 µm. (1.51 MB TIF) [file pone.0013003.s006.tif]
